# Predictors of in-hospital mortality in diabetic patients with non-ST-elevation myocardial infarction

**DOI:** 10.1186/s43044-022-00256-y

**Published:** 2022-03-28

**Authors:** Seyyed Mojtaba Ghorashi, Mojtaba Salarifar, Hamidreza Poorhosseini, Saead Sadeghian, Arash Jalali, Hassan Aghajani, Ali-Mohammad Haji-Zeinali, Negar Omidi

**Affiliations:** 1 Department of Cardiovascular Disease Research, Tehran Heart Center, Tehran University of Medical Sciences, Tehran, Iran; 2 Department of Interventional Cardiology, Tehran Heart Center, Tehran University of Medical Sciences, Tehran, Iran

**Keywords:** Myocardial infarction, Mortality, Diabetes mellitus, Ejection fraction

## Abstract

**Background:**

There have been little data about the additive effects of coronary risk factors on mortality in diabetic patients presenting with non-ST-segment elevation myocardial infarction (NSTEMI). This study aimed to evaluate the predictors of mortality in diabetic patients presenting with NSTEMI. All patients admitted to Tehran Heart Center (THC) with a confirmed diagnosis of NSTEMI and a history of diabetes mellitus (DM) type 2 between September 2003 and April 2017 were included. Clinical characteristics and paraclinical data such as lipid profiles, creatinine, hemoglobin, and hemoglobin A1C (HbA1C) were evaluated in these patients to predict in-hospital mortality. The approach for model calibration was a logistic regression with the backward elimination method.

**Results:**

Of a total of 9158 patients with non-ST-segment elevation myocardial infarction, 3133 had diabetes mellitus type 2 and met our criteria to enter the final analysis. In the multivariable analysis, age, chronic obstructive pulmonary disease, and a severely reduced left ventricular ejection fraction (LVEF) significantly increased the rate of in-hospital mortality, whereas mildly and moderately reduced left ventricular ejection fraction did not increase the rate of mortality.

**Conclusions:**

Age, chronic obstructive pulmonary disease (COPD), and severely reduced LVEF (< 30%) independently increased in-hospital mortality in our diabetic patients with a confirmed diagnosis of NSTEMI. Severely reduced LVEF had the strongest relationship with in-hospital mortality, whereas the mean HbA1C level and the type of DM management exerted no significant effect on in-hospital mortality.

## Background

The most frequent cause of mortality and morbidity is cardiovascular disease (CVD), [1] and the greatest burden of diseases worldwide belongs to ischemic heart disease [2]. Coronary artery disease (CAD) is a common condition that affects several million adults. Despite a reduction in the CVD rate in developed countries, ischemic heart disease is still high in developing countries. Acute myocardial infarction (MI) encompasses ST-segment elevation myocardial infarction (STEMI) and non-ST-segment elevation myocardial infarction (NSTEMI). Although in-hospital mortality is higher in SETMI than NSTEMI, the chance of 30 days and 1-year mortality is higher in NSTEMI compared to STEMI [3]. The global prevalence of diabetes mellitus (DM) in 2019 is estimated to be 9.3%, rising to 10.2% by 2030 and 10.9% (700 million) by 2045 [4]. DM is an established risk factor for CAD [5]. Moreover, diabetic patients with MI have adverse cardiovascular effects and higher in-hospital morbidity and mortality rates [6]. Previous studies have focused on mortality in patients with NSTEMI and indicated that DM is one of the major risk factors [7]. Nonetheless, the additive effects of risk factors in diabetic patients with MI have yet to be fully elucidated, and so do the factors influencing the mortality of MI in diabetic patients. Accordingly, in the present study, we sought to determine these specific factors in diabetic patients presenting with NSTEMI, which, to the best of our knowledge, has not been assessed so far.

## Methods

### Data source

This study was performed on patients admitted to Tehran Heart Center (THC) with a confirmed diagnosis of NSTEMI between September 2003 and April 2017 using the THC Acute Coronary Syndrome Registry. The final analysis was conducted only on patients with a history of type 2 DM.

### Study population

Of a total of 9158 patients, 3133 patients were qualified to enter our study. For the purposes of predicting in-hospital mortality, information was gathered on baseline characteristics (age and sex), major coronary risk factors (hypertension, dyslipidemia, cigarette smoking, opium abuse, and family history), past medical history (previous MI, coronary artery bypass graft, and chronic obstructive pulmonary disease (COPD)) type of DM treatment (diet, oral medication, and insulin or combination therapy), laboratory tests (HbA1C), high-density lipoprotein, low-density lipoprotein, total cholesterol, triglyceride, creatinine, and hemoglobin), LVEF on admission, and drug history (aspirin, clopidogrel, statins, angiotensin-converting enzyme inhibitors (ACEIs)/angiotensin II receptor blockers (ARBs)).

### Ethical statement

The study protocol was approved by the Ethics Committee of Tehran University of Medical Sciences, and it conforms to the ethical guidelines of the 2013 Declaration of Helsinki.

### Definitions and study endpoints

NSTEMI was defined as non-persistent ST-segment elevation in patients with acute chest pain and cardiomyocyte necrosis (the detection of an increase and/or decrease in a cardiac biomarker) [8]. DM was defined as either a fasting blood glucose level of more than 126 mg/dL or a history of prescribed medications [9]. Hypertension was defined as blood pressure more than 140/90 mm Hg or the use of antihypertensive medications [10]. Dyslipidemia was defined as a minimum total cholesterol level of 240 mg/dL, a minimum triglyceride level of 200 mg/dL, a high-density lipoprotein level of less than 40 mg/dL in men and less than 50 mg/dL in women, a minimum low-density lipoprotein level of 160 mg/dL, or a history of prescribed lipid-lowering medications [11]. The body mass index was defined as a weight-to-height ratio, calculated by dividing weight (kg) by the square of height (m); and a minimum index of 30 kg/m^2^ was defined as obesity [12]. Cigarette smoking and opium consumption status was defined as a current user or a daily user, a former user who had quit at least three months previously, and a never user based on their self-report [13]. COPD was defined as a chronic condition with abnormal pulmonary function (post-bronchodilator forced expiratory volume in one second (FEV1)/forced vital capacity < 0.70 and FEV1 < 80% with frequent exacerbation, necessitating recurrent hospitalization and the long-term use of bronchodilators) [14]. The LVEF (determined by echocardiography) was categorized as normal (50–70%), mildly reduced (40–49%), moderately reduced (30–39%), and severely reduced (< 30%) [15]. Chronic kidney disease (CKD) or renal failure was defined based on glomerular filtration rate (GFR < 60 ml/min per 1.73 m^2^) for ≥ 3 months [16].

### Statistical analysis

The univariate effects of the variables on mortality were evaluated applying a logistic regression model, and the effects were reported through odds ratios (OR) with 95% confidence intervals (CIs). Variables with a *P* value of less than 0.05 in the univariate analyses were candidates to enter the final model. A logistic regression model with the backward elimination method (with 0.05 and 0.1 as entry and removal probabilities) was used to detect the multiple predictors of mortality. A complete-case analysis was conducted. The discrimination power of the final model was assessed using the area under the receiver operating characteristic curve. Model calibration was evaluated using the Hosmer–Lemeshow goodness of fit test. All the statistical analyses were conducted with IBM SPSS Statistics for Windows, version 23.0 (Armonk, NY: IBM Corp).

## Results

### Population

In total, of 9158 patients with a confirmed diagnosis of NSTEMI between September 2003 and April 2017 in THC, 3133 patients had type 2 DM and were enrolled in the final analysis. The baseline characteristics of the patients according to their discharge conditions are demonstrated in Table 1. The study population (mean age of 64.7 ± 10.59 years) was comprised of mostly men (57%). The most frequent conventional risk factor was cigarette smoking (75.8%). The most common DM management strategy was an only oral antihyperglycemic agent (59.9%), followed by only insulin (20.9%). Finally, 117 (3.7%) patients died within the hospital admission period that 47% of them were women (55/117).

**Table 1:** Baseline characteristics of the study population and univariable effects on mortality

Characteristic	Total	Alive	Dead	OR	95% CI		*P* value
					Lower	Upper	
Age (y), mean (SD)	64.7 (10.59)	64.5 (10.53)	69.8 (10.9)	1.05	1.03	1.07	0.001
Sex (male), n (%)	1787 (57.0)	1725(57.2)	62 (53.0)	0.84	0.58	1.22	0.368
Hypertension, n (%)	2200 (70.2)	2130 (70.6)	70 (59.8)	0.62	0.42	0.90	0.013
Dyslipidemia, n (%)	2020 (64.5)	1954 (64.9)	66 (56.4)	0.70	0.48	1.01	0.062
Family history, n (%)	261 (8.5)	255 (8.6)	6 (5.5)	0.61	0.26	1.40	0.247
COPD, n (%)	44 (1.4)	40 (1.3)	4 (3.4)	2.63	0.92	7.48	0.070
CABG, n (%)	553 (17.7)	532 (17.7)	21 (17.9)	1.02	0.63	1.65	0.934
Previous MI, n (%)	524 (17.0)	504 (16.9)	20 (17.2)	1.02	0.62	1.66	0.933
RF, n (%)	317(10.1)	289 (9.6)	28 (23.9)	2.96	1.90	4.61	< 0.001
LVEF, n (%)							
Normal	470 (18.4)	463 (18.8)	7 (7.4)				< 0.001
Mild reduction	459 (18.0)	540 (18.3)	9 (9.6)	1.32	0.48	3.58	0.582
Moderate reduction	1044 (40.8)	1013 (41.1)	31 (33.0)	2.02	0.88	4.63	0.095
Severe reduction	583 (22.8)	536 (21.8)	47 (50.0)	5.80	2.59	12.95	< 0.001
ACEI/ARB, n (%)	1356 (45.7)	1313 (46.0)	43 (37.7)	0.71	0.48	1.04	0.084
Aspirin, n (%)	2239 (95.8)	2197 (95.9)	42 (93.3)	0.60	0.18	1.98	0.408
Clopidogrel, n (%)	1108 (85.6)	1087 (85.8)	21 (77.8)	0.58	0.23	1.45	0.246
Statin, n (%)	1920 (88.0)	1886 (88)	34 (82.9)	0.65	0.28	1.50	0.322
Body mass index (kg/m^2^), n (%)	27.9 (4.65)	27.9 (4.63)	28.3 (5.97)	1.01	0.95	1.08	0.615
Cigarette smoking, n (%)							
Current	2350 (75.8)	2255 (75.6)	95 (981.2)				0.320
Former	294 (75.8)	287 (9.6)	7 (6.0)	0.57	0.26	1.26	0.168
Never	455 (14.7)	440 (14.8)	15 912.8)	0.80	0.46	1.40	0.454
Opium, n (%)							
Current	2897 (92.5)	2784 (92.4)	113 (96.6)				0.403
Former	33 (1.1)	33 (1.1)	0 (0.0)				0.998
Never	201 (6.4)	197 (6.5)	4 (3.4)	0.50	0.18	1.37	0.178
Diabetes treatment, n (%)							
Diet	174 (5.6)	169 (5.7)	5 (4.6)	0.55	0.19	1.59	0.275
Oral	1854 (59.9)	1805 (60.5)	49 (45.4)	0.51	0.27	0.95	0.036
Insulin	674 (20.9)	613 (20.5)	34 (31.5)	1.04	0.54	2.01	0.895
Combination	160 (5.2)	153 (5.1)	79 (6.5)	0.86	0.33	2.20	0.757
Total cholesterol, median	165 (134, 198)	165 (134, 198)	160.5 (133, 204)	0.9	0.99	1.00	0.717
Triglyceride, median	149 (106, 213)	149 (106, 213)	137.5 (94, 183)	0.99	0.99	1.00	0.267
HDL, median	38.5 (10.57)	38.5 (10.58)	36.8 (10.14)	0.98	0.95	1.01	0.321
LDL, median	99 (74, 126)	99 (74, 126)	99 (70, 127)	1.00	0.99	1.00	0.943
Creatinine (mg/dL), median	1.0 (0.8, 1.2)	1.0 (0.8, 1.2)	1.1 (0.8, 1.6)	1.20	0.99	1.45	0.051
HbA1C, n (%)	8.2 (7.1, 9.5)	8.8 (7.2, 10.1)	1.135	0.94	1.36	0.18	0.178
Hemoglobin (mg/dL), n (%)	13.8 (2.03)	13.8 (2.03)	13.2 (2.01)	0.86	0.73	1.02	0.098

Continuous variables are presented as the mean (SD) or the median (25th and 75th percentiles)

Categorical variables are described as frequencies (percentages); n (%)

*COPD* chronic obstructive pulmonary disease, *CABG* coronary artery bypass graft, *MI* myocardial infarction, *RF* renal failure, *LVEF* left ventricular ejection fraction, *ACEI* angiotensin-converting enzyme inhibitor, *ARB* angiotensin II receptor blocker, *HDL* high-density lipoprotein, *LDL* low-density lipoprotein

### Univariate analysis

Age, renal failure, and severely reduced LVEF showed statistically significant differences between the surviving and non-surviving patients (Table 1). Dyslipidemia, cigarette smoking, and opium consumption had no significant effect on in-hospital mortality. Neither drug history of antiplatelet and statin nor a previous history of coronary artery bypass graft surgery and MI significantly differed between surviving and non-surviving patients.

### Multivariate analysis

**Fig. 1:**
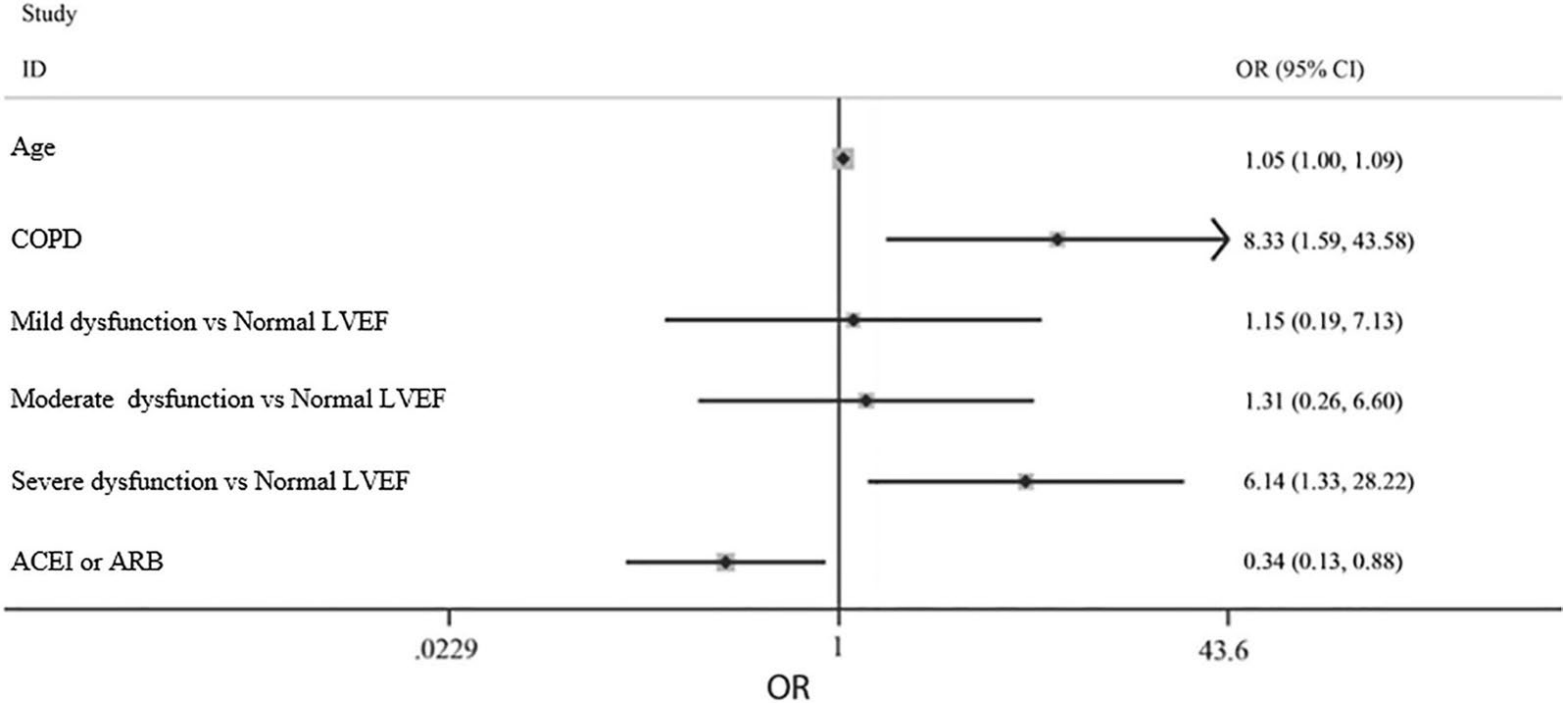
Forest plot scheme in the multivariable model. *P* value for the Hosmer–Lemeshow test: 0.612. *ACEI* Angiotensin-converting-enzyme inhibitor, *ARB* Angiotensin II receptor blocker, *COPD* Chronic obstructive pulmonary disease, *LVEF* Left ventricular ejection fraction

In the multivariable analysis with the backward elimination method, among all the variables, age, COPD, LVEF, and ACEI/ARBs consumption were identified as the independent predictors of in-hospital mortality (Table 2). The strongest predictor was severely reduced LVEF (< 30%) (OR: 6.137, 95% CI: 1.335–28.221; *P* = 0.020). Although COPD had a higher OR (OR: 8.33, 95% CI: 1.59–43.58; *P* = 0.012) with respect to the prevalence in our population, its power decreased. The Forest plot (Fig. 1) displays the independent impact of each predictor. As is illustrated in the plot, the use of ACEIs or AT2R blockers was protective against mortality.

**Table 2:** Multivariable analysis of the risk factors predicting mortality

Variable	OR	95% CI		*P* value
		Lower	Upper	
Age	1.046	1.001	1.092	0.046
COPD	8.329	1.592	43.580	0.012
LVEF				0.003
Mildly reduced	1.153	0.186	7.127	0.879
Moderately reduced	1.306	0.258	6.596	0.747
Severely reduced	6.137	1.335	28.221	0.020
ACEI or ARB	0.335	0.128	0.877	0.026

*COPD* chronic obstructive pulmonary disease, *LVEF* left ventricular ejection fraction, *ACEI* angiotensin-converting enzyme inhibitor, *ARB* angiotensin II receptor blocker

## Discussion

Patients with DM are more susceptible to acute coronary syndrome and have higher risks for in-hospital mortality and major adverse cardiovascular events than are patients without DM [6, 17]. In this study, we aimed to evaluate the predictors associated with in-hospital mortality in type 2 diabetic patients with confirmed NSTEMI.

According to our univariable analysis, diabetic patients who were managed by only oral antihyperglycemic agents had a better outcome compared with ones treated by only insulin or combination therapy. Based on previous studies, it appears that non-insulin-treated DM is associated with better DM control and better outcomes than insulin-treated ones. Non-insulin medications used in the DM treatment reduced the incidence of cardiovascular events in diabetic patients and have a beneficial effect on MI in follow-up [18]. In a study by Noman et al. [19] on early mortality following percutaneous coronary intervention (PCI) in diabetic patients, higher mortality occurred in insulin-treated patients.

CKD is an independent predictor of cardiovascular morbidity and mortality [20]. We have seen that renal failure is associated with higher in-hospital mortality only in the univariable study. In addition, the higher creatinine level had a trend to a worse outcome. Wang et al. [21], in a study regarding the impact of CKD on clinical outcomes in NSTEMI receiving PCI, found that in-hospital mortality does not significantly differ between patients with and without advanced CKD. However, long-term follow-up of CKD patients consistently reveals poor outcomes. Therefore, the presence of CKD should be taken into consideration in the management of MI and warrants further research.

Another independent risk factor for in-hospital mortality was COPD. The COPD prevalence among study participants was very low (1.4%), and the COPD prevalence in patients who died was 3.4% (4/117). Zhang et al. [22] evaluated patients who underwent PCI and concluded that a history of COPD had a strong association with adverse cardiac events and increased risk of all-cause mortality, which attributed to initial lower baseline LVEF and higher prevalence of coronary risk factors. Agarwal et al. [23], in a study regarding the impact of COPD on patients with STEMI, found an increased risk of in-hospital mortality among these patients and in their STEMI patients with a history of DM. Moreover, they conveyed the point that a positive correlation between COPD and in-hospital mortality is due to the increased risk of new-onset heart failure, acute respiratory failure, and cardiogenic shock in those patients. Another study by Enriquez et al. [24] demonstrated that in patients presenting with NSTEMI, a history of chronic lung disease was independently associated with a higher risk of in-hospital mortality. Two reasons would be enumerated for this association, including an elevated risk of bleeding and more variable approaches to care of NSTEMI because of the individual physician and system decisions that cause lower and delayed revascularization in NSTEMI patients. Still, further research is needed to investigate the predictive power of COPD for mortality in each subtype of MI.

Our results revealed no statistically significant difference between gender and mortality. In line with our findings, Wang et al. [25] conducted an exhaustive meta-analysis evaluating the possible role of gender in the mortality rate in the short and long term after NSTEMI. They observed no significant differences between men and women in the prognosis of NSTEMI. Further research is recommended on the impact of sex on mortality in diabetic patients with NSTEMI.

Age increased in-hospital mortality in our diabetic patients with NSTEMI as an independent factor. According to McNamara et al. [26] study using ACTION (Acute Coronary Treatment and Intervention Outcomes Network) Registry–GWTG (Get With the Guidelines) database, increasing age was associated with in-hospital mortality.

Even though the mean HbA1C level was above the normal level in our study, it had no statistically significant relationship with in-hospital mortality. Liang et al. [27] studied patients with MI in China and revealed that HbA1C had no significant effect on mortality.

Our study showed that severely reduced LVEF is a robust predictor of in-hospital mortality. This finding has two implications. Firstly, mortality does not increase significantly until the LVEF drops to less than 30%, and secondly, a mildly or moderately reduced LVEF has no significant correlation with in-hospital mortality. Chehab et al. [28] demonstrated that patients with acute MI and reduced EF (< 35%) developed higher in-hospital mortality. According to Emet et al.’s [29] studies in Istanbul, there was a significant association between mean LVEF and in-hospital mortality.

As indicated in our study, the univariate analysis demonstrated that hypertension had a protective effect against in-hospital mortality although this effect was not observed in multivariable analysis. It gives the impression that using ACEIs/ARBs was the major cause of protective effects, not hypertension. History of ACEIs/ARBs usage conferred a protective effect against in-hospital mortality among diabetic patients with NSTEMI. Zuanetti et al. [30] conducted a study on diabetic patients with suspected Acute MI and showed that early treatment with ACEIs caused a reduction in 6-week mortality. Niskanen et al. [31] studied patients with hypertension and showed that ACEIs diminished mortality and cardiovascular events in those with DM. According to previous studies, this finding might be because of the efficacy of ACEIs in ameliorating the enlargement of the ventricles and preventing decrements in ventricular function [32].

We used paper or electronic documented data and databases and, as a result, encountered some missing values. We had no mid-term and long-term follow-ups of our patients is another weakness of note. We recommend that future investigations evaluate long-term outcomes in relation to risk factors and their prognosis.

## Conclusions

Age and severely reduced systolic function constituted the independent predictive factors of mortality in our diabetic patients presenting with NSTEMI. Severely reduced LVEF (< 30%) had the strongest relationship with in-hospital mortality, whereas the mean HbA1C level and the type of treatment (either oral hypoglycemic agents or insulin) exerted no significant effect on in-hospital mortality. Furthermore, ACEIs/ARBs in drug history conferred protective effects in our diabetic patients with NSTEMI.

## Data Availability

The datasets analyzed during the current study are not publicly available due to the institutional policy but are available from the corresponding author on reasonable request.

## Abbreviations

ACEI: Angiotensin-converting enzyme inhibitor
ARB: Angiotensin II receptor blocker
CAD: Coronary artery disease
CI: Confidence interval
CKD: Chronic kidney disease
COPD: Chronic obstructive pulmonary disease
CVD: Cardiovascular disease
DM: Diabetes mellitus
FEV1: Forced expiratory volume in 1s
GFR: Glomerular filtration rate
HbA1C: Hemoglobin A1C
LVEF: Left ventricular ejection fraction
NSTEMI: Non-ST-segment elevation myocardial infarction
STEMI: ST-segment elevation myocardial infarction
THC: Tehran Heart Center
